# Cell Migration Is Regulated by AGE-RAGE Interaction in Human Oral Cancer Cells *In Vitro*

**DOI:** 10.1371/journal.pone.0110542

**Published:** 2014-10-16

**Authors:** Shun-Yao Ko, Hshin-An Ko, Tzong-Ming Shieh, Weng-Cheng Chang, Hong-I Chen, Shu-Shing Chang, I-Hsuan Lin

**Affiliations:** 1 Graduate Institute of Medical Science, College of Health Science, Chang Jung Christian University, Tainan, Taiwan; 2 Innovate Research Center of Medicine, Chang Jung Christian University, Tainan, Taiwan; 3 Department of Dental Hygiene, China Medical University, Taichung, Taiwan; University of Miami, United States of America

## Abstract

Advanced glycation end products (AGEs) are produced in an irreversible non-enzymatic reaction of carbohydrates and proteins. Patients with diabetes mellitus (DM) are known to have elevated AGE levels, which is viewed as a risk factor of diabetes-related complications. In a clinical setting, it has been shown that patients with oral cancer in conjunction with DM have a higher likelihood of cancer metastasis and lower cancer survival rates. AGE-RAGE (a receptor of AGEs) is also correlated with metastasis and angiogenesis. Recent studies have suggested that the malignancy of cancer may be enhanced by glyceraldehyde-derived AGEs; however, the underlying mechanism remains unclear. This study examined the apparently close correlation between AGE-RAGE and the malignancy of SAS oral cancer cell line. In this study, AGEs increased ERK phosphorylation, enhanced cell migration, and promoted the expression of RAGE, MMP2, and MMP9. Using PD98059, RAGE antibody, and RAGE RNAi to block RAGE pathway resulted in the inhibition of ERK phosphorylation. Cell migration, MMP2 and MMP9 expression were also reduced by this treatment. Our findings demonstrate the importance of AGE-RAGE with regard to the malignancy of oral cancer, and help to explain the poor prognosis of DM subjects with oral cancer.

## Introduction

Advanced glycation end products (AGEs) are the result of a Maillard reaction between carbohydrates and proteins. Aging and reduced metabolic function have been shown to increase the formation of AGEs [1]–[5]. Increased AGE accumulation has also been observed among patients with Alzheimer's disease (AD) or diabetes mellitus (DM) [6]–[8]. Moreover, AGEs have been shown to play a role in the pathogenesis of DM, such that AGE accumulation is regarded as a risk factor for various complications of the disease [9]–[14].

Recent studies have demonstrated that AGEs and RAGEs (receptors of AGEs) regulate cell migration [15]–[17]. RAGE overexpression has been linked to cancers of the colon, larynx, tongue, stomach, and mouth [18]–[20], through carcinogenesis [21], cell proliferation, metastasis, invasion, and angiogenesis [22]–[25]. In a clinical study that included patients suffering from oral cancer as well as DM, cancer became increasingly invasive, resulting in a decline in survival rates [26]. That study further identified a correlation between oral cancer and DM [27]. RAGE has been shown to be closely associated with invasion in oral cancer [15], and the malignancy of cancer can be enhanced by glyceraldehyde-derived AGEs [17]. However, the underlying mechanism responsible for these effects remains unclear.

This study investigated the apparently strong correlation between AGEs and the malignancy of oral cancer. Our results demonstrate that AGEs enhance cell migration and also increase ERK phosphorylation and the expression of RAGE, MMP2, and MMP9. Pretreatment with PD98059 (an ERK inhibitor) was found to suppress the effects of AGEs; however, RAGE expression was not inhibited. RAGE antibodies were also used to block the conjugation of AGEs, which resulted in reduced ERK phosphorylation, the expression of MMP2, MMP9, and cell migration. Furthermore, RAGE RNAi appears to regulate these effects, suggesting that the AGE-RAGE system alters cell migration through ERK phosphorylation and the regulation of downstream pathways. Our findings provide further proof that AGE-RAGE plays an important role in determining the malignancy of oral cancer.

## Materials and Methods

### Reagents

Phenylmethylsulfonyl fluorides (PMSF), bovine serum albumin (BSA), DL-Glyceraldehyde, and PD98059 were purchased from Sigma (St. Louis, MO, USA). DMEM media, fetal bovine serum (FBS), penicillin, streptomycin, Hanks Balanced Salt Solution (HBSS), trypan blue, and Lipofectamine RNAiMAX were purchased from Invitrogen (Carlsbad, CA, USA). GAPDH (Cat No.: MAB374) was purchased from Chemicon (Temecula, CA, USA). ERK (Cat No.: SC-94), p-ERK (Cat No.: SC-7383), RAGE (Cat No.: SC-94), and RAGE siRNA (Cat No.: SC-36374) were purchased from Santa Cruz biotechnology (Santa Cruz, CA, USA). MMP2 (Cat No.: 2763-S) and MMP9 (Cat No.: 2551-S) were purchased from Epitomics (Burlingame, CA, USA). Nitrocellulose membranes were purchased from PALL corp. (Ann Arbor, MI, USA). Enhanced chemiluminescence (ECL) was purchased from Millipore (Billerica, MA, USA). Wst-1 kit was purchased from Clontech Laboratories, Inc. (Mountain View, CA, USA). Culture-Insert was purchased from ibidi (Verona, WI, USA).

### Preparation of AGEs

AGEs were prepared by incubating BSA (pH = 7.4) in PBS with 20 mM DL-Glyceraldehyde at 37°C for 1 week. The product was dialyzed in PBS at 4°C for 2 hours, and this cycle was repeated 5 times. The product was then concentrated at 4°C using an Amicon Ultra protein concentration tube (Millipore) and centrifuged at 3,000 rpm for 30 minutes before being stored at −80°C [28].

### Cell culture and treatment

The oral cancer cell line SAS (Japanese Collection of Research Bioresources Cell Bank [JCRB], Japan) was grown at 37°C under a 5% CO_2_ atmosphere. The culture was maintained in DMEM media (Invitrogen) routinely supplemented with 10% FBS, 100 units/ml of penicillin, 2mM L-glutamine, and 100 µg/ml streptomycin. Cells were incubated serum-free for 24 hours prior to treatment.

### Trypan blue dye exclusion assay

Cells were seeded on 6-well plates at a concentration of 10^5^ per well and cultured for 24 hours. Cell viability was determined according to their ability to exclude 0.5% trypan blue (Invitrogen) following treatment with 100–400 µg/ml AGEs for 24 and 48 hours, respectively. An equal volume of trypan blue dye solution (0.1%, w/v), HBSS, and cell slurry were combined and maintained at room temperature for five minutes. Samples were then loaded onto a hemocytometer to categorize cells as living or dead according to the uptake of dye. All figures presented in this study are the mean values of triplicate experiments.

### WST-1 assay

Cells were seeded on 96-well plates at a concentration of 5×10^3^ per well and cultured for 24 hours. Cell proliferation was detected by the WST-1 kit (Clontech) in conditioned medium. Samples were prepared according to the protocol outlined by the manufacturer. Briefly, following treatment with AGEs (0–400 µg/ml), the conditioned medium was centrifuged at 2,000 rpm for 10 minutes. Supernatant was then transferred into 96 well plates (100 µl/well). Reaction Mixture (100 µl) was added to each well, followed by incubation for 30 minutes. During the incubation period, samples were maintained at room temperature (RT) and protected from light. Absorbance of the samples was measured at 490 nm. Reference wavelength should be greater than 600 nm. All figures presented in this study are the mean values from experiments performed in triplicate.

### RNA Interference Experiments

Cells were seeded on 6-well plates at a concentration of 2×10^5^ cells per well and cultured for 24 hours. The cells were then transfected with RAGE siRNA (a pool of 3 target-specific 19–25 nt siRNA; Santa Cruz) or the sense strand sequence of the RNAi as a negative control (5′-UUCUCCGCCCGUGUCACGU-3′) [29]. Transfections were conducted using Lipofectamine RNAiMAX (Invitrogen) according to manufacturer's protocol. Specifically, dilute 40 pmol of RNAi duplex in 250 µl Opti-MEM I reduced serum medium without serum. We then gently mix the Lipofectamine RNAiMAX and diluted 4 µl of the mixture into 50 µl Opti-MEM I reduced serum medium. The dilute RNAi duplex was combined with dilute Lipofectamine RNAiMAX and incubated for 20 minutes at room temperature. Finally, the RNAi duplex- Lipofectamine RNAiMAX complexes were added to each well. This resulted in a final volume of 600 µl and a final siRNA concentration of 20 nM. Cells were incubated for 48 hours prior to being used in experiments.

### Western blot

Proteins (30 µg) were resolved using 10% SDS-PAGE gel and then transferred onto nitrocellulose membranes (PALL Corp.). The membranes were blocked using non-fat milk and incubated overnight at 4°C with the following primary antibodies: anti-p-ERK (dilution of 1∶1,000); anti-ERK (dilution of 1∶1,000); anti-MMP2 (dilution of 1∶1,000); anti-MMP9 (dilution of 1∶1,000); anti-RAGE (dilution of 1∶1,000; anti-GAPDH (dilution of 1∶40,000). Primary antibodies were then removed and membranes were washed with PBST buffer for 30 minutes. The membranes were subsequently incubated for 45 minutes at RT with the following secondary antibodies: horseradish peroxidase conjugated anti-mouse (dilution of 1∶4,000) and anti-rabbit (dilution of 1∶4,000) (Chemicon). Finally, secondary antibodies were removed and the membranes were washed with PBST buffer twice for 30 minutes. Signals were detected using the Western Lighting Chemiluminescence Reagent Plus kit (Millipore). The densities of the signals were measured using an imaging system, and the signals were quantified after being normalized against those of GAPDH. All figures presented in this paper are the mean values from experiments performed in triplicate.

### Cell migration assay

Cells were seeded on 6-well plates using Culture-Insert (ibidi) at a concentration of 6×10^5^ cells per well and then cultured for 24 hours. After a period of 24 hours in serum-free conditions, the Culture-Inserts were removed and cells were washed using HBSS, before being treated with AGEs for analysis of cell migration.

### Zymorgraphy assay

Following treatment with 200–400 µg/ml AGEs for 4 or 24 hours, cells were seeded on 7 cm dishes at a concentration of 1×10^6^ cells. Zymography was used to detect the function of MMP2 and MMP9 in conditional medium. To achieve this, we used 7.5% SDS-PAGE gel containing 0.1% gelatin (J.T.Baker, Center Valley, PA, USA). The gel was then washed with 2.5% Triton X 100 for 30 minutes at RT. Triton X 100 was removed and gel was washed with RO H_2_O. We then added renaturing buffer (50mM Tris-HCl pH 7.2,20%glycerol) for 30 minutes at RT. Renaturing buffer was removed and gel was washed with RO H_2_O. We then added developing buffer (50mM Tris-HCl pH 8.8, 5mM CaCl2, 1mM ZnCl2) for 24 hours at 37°C. Developing buffer was removed and gel was washed with RO H_2_O. Gel staining was conducted for 1 hour at RT using PageBlue Protein Staining (Fermentas, Lafayette, CO, USA). Gel destaining was conducted using RO H_2_O at RT. (Fig S1).

### Statistical analyses

We conducted student t-tests, one-way ANOVA, and p values of <0.05 were considered statistically significant.

## Results

### Influence of AGEs on oral cancer cells

**Figure 1:**
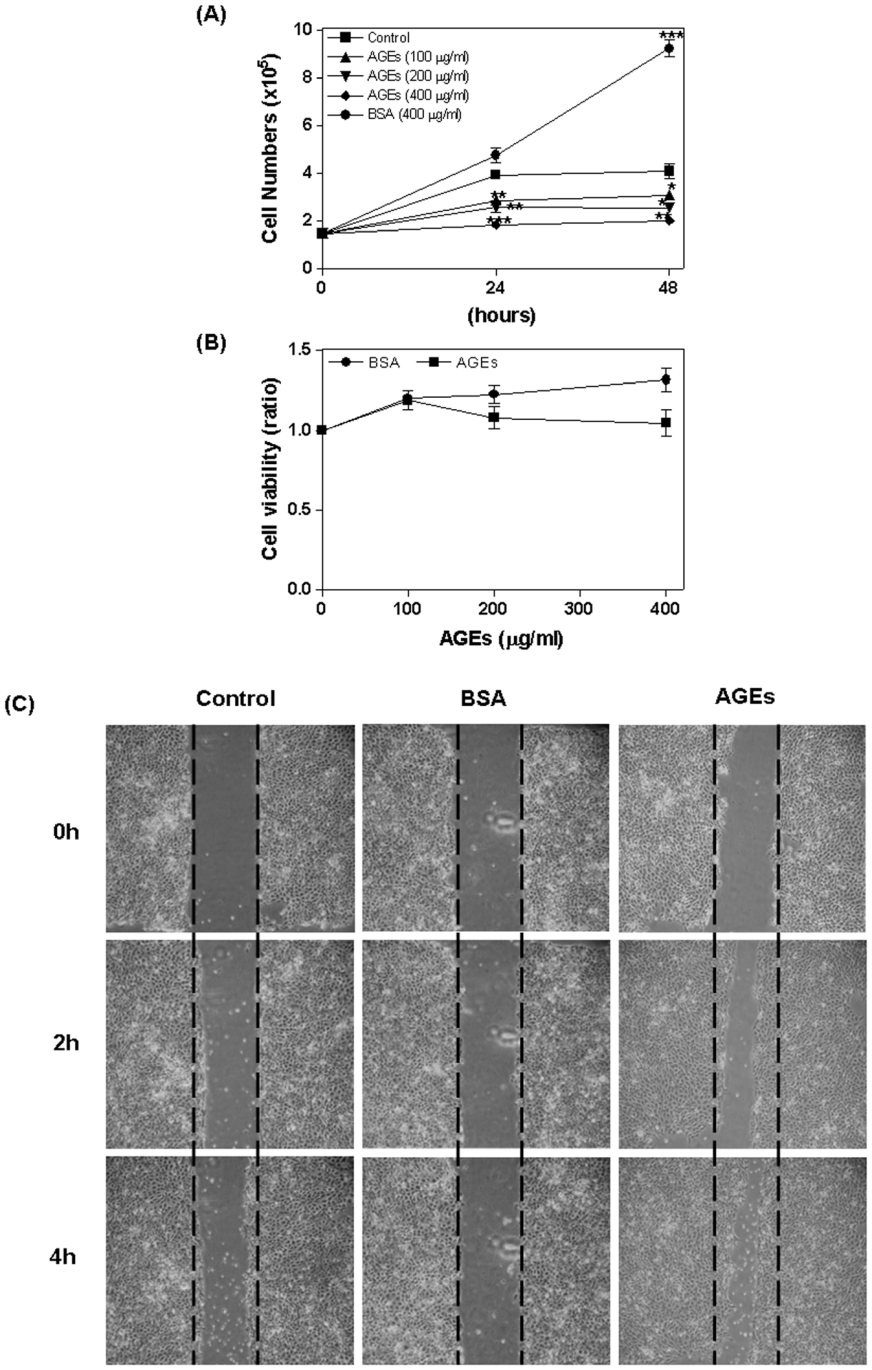
Inhibition of cell proliferation by AGEs. SAS cells were treated with AGEs (0–400 µg/ml) or BSA (0–400 µg/ml) for 24 to 48 hours. The number of cells and proliferation were detected using trypan blue dye exclusion (A) and a WST-1 assay (B). AGEs significantly reduced the number of cells; BSA (as a negative control) increased viability (A). AGEs also inhibited cell proliferation (B). Treatment with AGEs (400 µg/ml; 0–4 hours) enhanced cell migration, while treatment with BSA did not (C).

We first assessed the influence of AGEs on cell viability. SAS cells were treated with AGEs (0–400 µg/ml) or BSA (0–400 µg/ml) for 24 or 48 hours, whereupon the number of cells and proliferation rate were determined according to trypan blue dye exclusion (Fig. 1A) and WST-1 assays (Fig. 1B). Compared with control cells, AGE treated cells showed a significant reduction in number of cells (24 hours AGEs 100: 2.88±0.16, *P* = 0.006; AGEs 200: 2.6±0.25, *P* = 0.008; AGEs 400: 1.85±0.05, *P* <0.0001; 48 hours AGEs 100: 3.1±0.18, *P* = 0.05; AGEs 200: 2.57±0.17, *P* = 0.01; AGEs 400: 2.03±0.08, *P* = 0.003). In contrast, BSA was shown to increase the number of cells (as a negative control, 24 hours: 4.77±0.32, NS; 48 hours: 9.25±0.35, *P* = 0.0004) (Fig. 1A). In addition, cell proliferation was shown to be inhibited by AGEs (Fig. 1B). Finally, treating cells with AGEs (400 µg/ml; 0–4 hours) enhanced migration; however, BSA did not have any effect on migration (Fig. 1C).

### AGEs regulation of RAGE, MMP2, and MMP9

**Figure 2:**
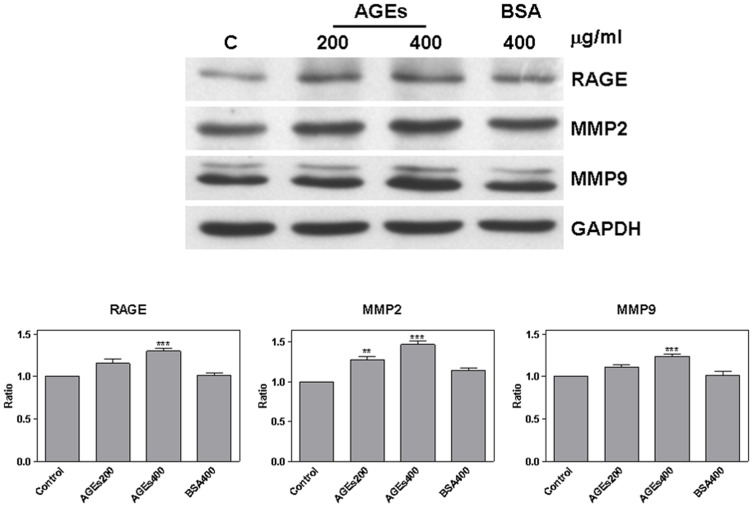
AGEs regulated RAGE, MMP2, and MMP9. After treating cells with AGEs (200 and 400 µg/ml) or BSA (400 µg/ml) for 24 hours, RAGE, MMP2, and MMP9 were detected by western blot analysis (A). AGEs significantly increased the expression of RAGE, MMP2, and MMP9 (B).

Cells were treated with AGEs (200 and 400 µg/ml) or BSA (400 µg/ml) for 24 hours. We then used western blot analysis to detect RAGE, MMP2, and MMP9. Compared with control cells, the result showed that AGEs treated cells presented a significant increase in RAGE (AGEs 400: 1.3±0.03, *P* = 0.0007), MMP2 (AGEs 200: 1.28±0.04, *P* = 0.002; AGEs 400: 1.47±0.04, *P* = 0.004), and MMP9 (AGEs 400: 1.24±0.03, *P* = 0.0008) (Fig. 2). Moreover, the functionality of MMP2 and MMP9 was increased after treatment with AGEs for 4 or 24 hours (Fig S2).

### Regulation of ERK phosphorylation by AGEs

**Figure 3:**
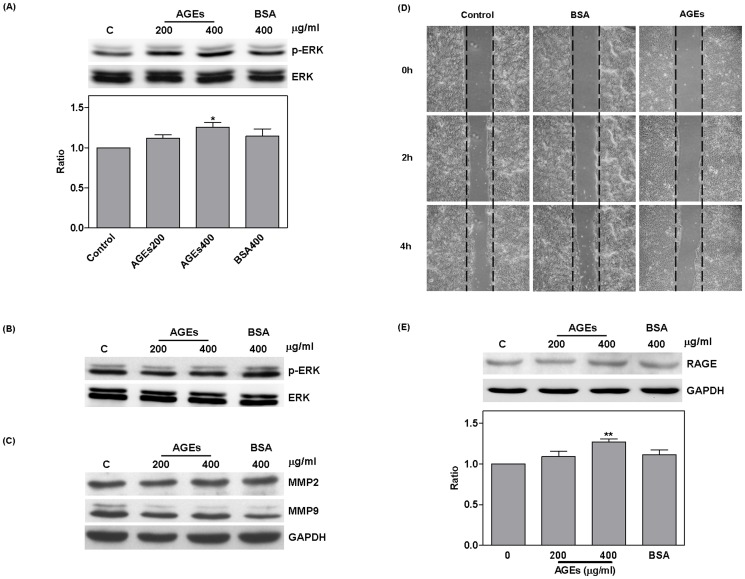
AGEs increased ERK phosphorylation. Western blot analysis showing that treating SAS cells with AGEs for 24 hours resulted in increased ERK phosphorylation (A). Pretreatment with PD98059 for 1 hour reduced ERK phosphorylation, MMP2 and MMP9 (B and C) as well as cell migration (D); however RAGE levels still increased (E).

In order to identify the pathway of AGEs, SAS cells were treated with AGEs or BSA for 24 hours. ERK phosphorylation was then detected using western blot analysis. Our results show that treatment with AGEs significantly increased ERK phosphorylation (AGEs 400: 1.26±0.06, *P* = 0.01) (Fig. 3A). ERK phosphorylation was enhanced following 4 hours AGEs treatment (Fig S2); however, pretreating cells with PD98059 for 1 hour resulted in a reduction in the expression of MMP2 and MMP9 (Fig. 3B and C). It appears that PD98059 pretreatment blocked the effects of AGEs on cell migration (Fig. 3D); however, RAGE expression was not affected (AGEs 400: 1.27±0.04, *P* = 0.003) (Fig. 3E).

### Regulation of ERK by AGEs via RAGE

**Figure 4:**
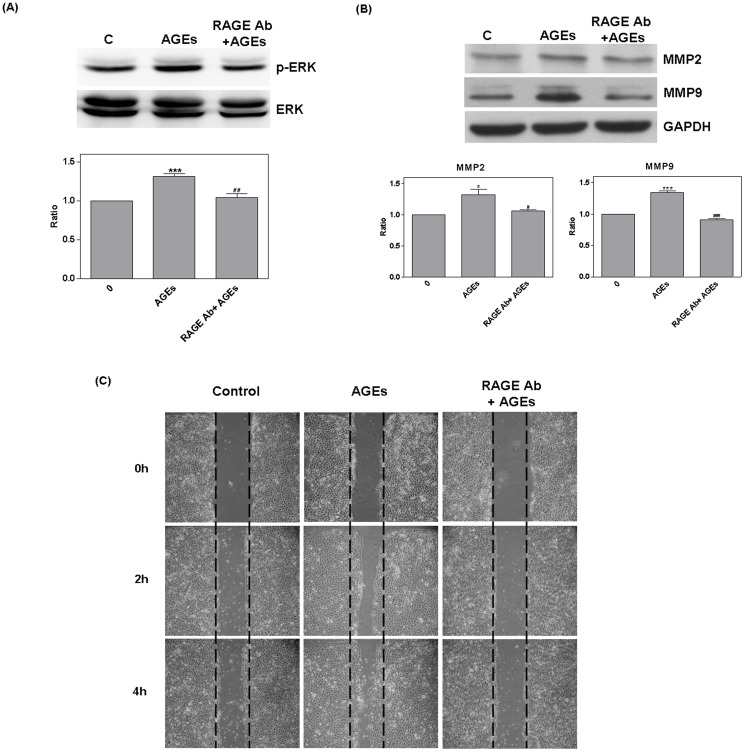
RAGE antibody blocked AGE regulation. The use of RAGE antibody (10 ng/ml pretreatment for 1 hour) to block AGE conjugation resulted in a significant increase in the ERK phosphorylation, MMP2, and MMP9 due to AGEs. Compared with AGEs treatment (#), RAGE antibody blocked ERK phosphorylation, MMP2, and MMP9 (A and B) as well as cell migration (C).

RAGE antibodies (10 ng/ml pretreatment for 1 hour) were used to block AGE conjugation. Treatment with AGEs resulted in a significant increase in ERK phosphorylation and the expression of MMP2, and MMP9 (ERK: 1.31±0.03, *P* = 0.0008; MMP2: 1.38±0.04, *P* = 0.0005; MMP9: 1.32±0.09, *P* = 0.02). Compared to cells treated with AGEs alone, cells treated with both AGEs and RAGE antibody demonstrated significantly inhibited ERK phosphorylation (*P* = 0.009) as well as reduced expression of MMP2 (*P* = 0.05), and MMP9 (*P* = 0.0003) (Fig. 4 A and B). RAGE antibodies were also shown to suppress cell migration (Fig. 4C).

**Figure 5:**
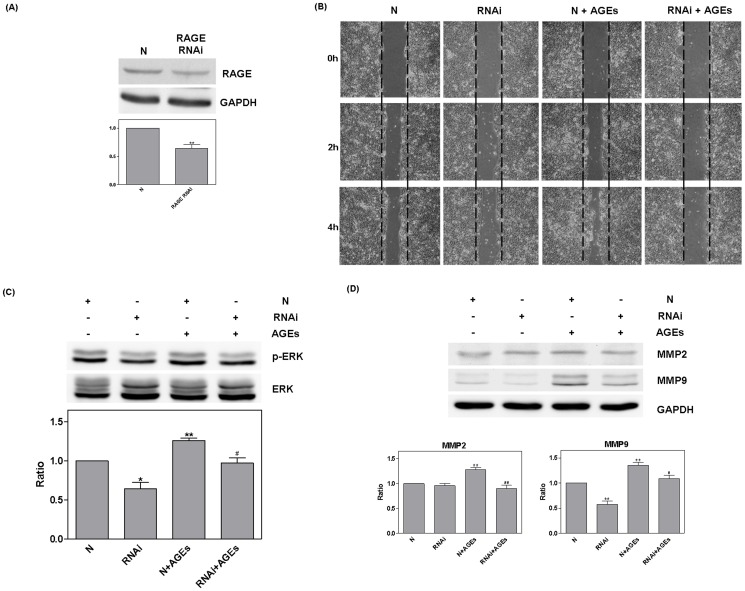
RAGE RNAi mediated influence of AGEs. RAGE RNAi (20 nM for 48 hours) was used to silence protein expression. Compared with the RNAi negative control (N), RAGE RNAi significantly reduced RAGE expression (A). The suppression of cell migration by RAGE RNAi occurred independently from treatment with AGEs (B). RAGE RNAi also inhibited ERK phosphorylation, MMP2, and MMP9. Compared with N+AGEs (#), RNAi +AGEs presented a significant reduction (C and D) and the negative control had no effect.

RAGE RNAi (20 nM for 48 hours) was then used to silence protein expression. Compared with the RNAi negative control (N), RAGE RNAi was shown to have a significantly suppress RAGE expression (0.64±0.07, *P* = 0.006) (Fig. 5A). The suppression of cell migration by RAGE RNAi was found to occur independently from the effects of AGEs (Fig. 5B). Furthermore, RAGE RNAi inhibited ERK phosphorylation (RNAi: 0.64±0.08, *P* = 0.01; N+AGEs: 1.26±0.03, *P* = 0.001) MMP2 (N+AGEs: 1.28±0.04, *P* = 0.003), and the secretion of MMP9 (RNAi: 0.58±0.06, *P* = 0.002; N+AGEs: 1.36±0.05, *P* = 0.003). Compared with N+AGEs, treatment with RNAi +AGEshad a significant effect on all three of these processes (ERK: *P* = 0.02; MMP2: *P* <0.003; MMP9: *P* = 0.04) (Fig. 5C and D). The negative control did not show significant effects.

## Discussion

A relationship between oral cancer and DM has been demonstrated by previous researchers [30]–[32]. Patients suffering from oral cancer in conjunction with DM are face with highly invasive cancer cells, and low survival rates [26]. However, the mechanism underlying the relationship between oral cancer and DM has not been elucidated. This is the first study to investigate a potential link between AGEs and oral cancer. We conducted this research to identify the role of the AGE-RAGE system in oral cancer, and to elucidate the relationship between oral cancer and DM.

Researchers have previously reported that AGEs and RAGE regulate cell migration [15]–[17], [33]. Our results confirm that AGEs enhance cell migration and also show that AGEs reduce cell viability. Takino et al. obtained similar findings using glyceraldehyde-derived AGEs [17]. Moreover, Bhawal et al. reported that RAGE is closely associated with the invasiveness of oral cancer [15], and our findings show that AGEs regulate cell migration via the expression of RAGE. In a clinical setting, the expression of MMP2 and MMP9 has appeared to be closely related to metastasis in oral cancer [34], [35]. AGEs have further been shown to increase the secretion of MMP2 [17] and regulate MMP9 expression [36], [37] via the ERK pathway [23], [38]. Our results support these earlier finding; specifically, that ERK phosphorylation and the expression of MMP2 and MMP9 are regulated by AGEs. Additionally, CD44 is a migration factor related to RAGE [39]. However, we failed to observed significant difference in CD44 expression following AGEs treatment (Data not shown). A similar report confirmed that the expression of CD44 is not affected by AGEs [40]. This suggests that AGEs-RAGE regulate cell migration via a pathway that is not CD44 dependent.

Recent studies have suggested that RAGE is a multiple receptor that regulates cell proliferation through its ligand (S100 or HMGB1) [41]–[43]. Xu et al. and Meghnani et al. also suggest that RAGE increases cell proliferation and related pathways [44], [45]. Moreover, HMGB1 has been found to regulate cell proliferation and metastasis via RAGE and melanoma inhibitory activity (MIA) pathway in oral cancer [46]. In our study, AGEs and BSA appear to differ in their effects on SAS cells. AGEs were shown to decrease the number of cells; however, BSA increased it. More specifically, in this study cells were incubated serum-free for 24 hours prior to treatment; thus BSA may have acted as a growth factor. Our finding that AGEs decreased the number of cells while increasing ERK phosphorylation clearly indicates that AGEs differ in their effects on cells. Valencia et al. also demonstrated that divergent pathways of gene expression are activated by the RAGE ligand S100 and AGEs [47]. Hence, the effects of AGE-RAGE system differ from those of other ligands.

The use of PD98059 to block the ERK pathway resulted in the suppression of cell migration and also reduced expression of MMP2 and MMP9; however, no difference was observed with regard to the influence of AGEs in increasing RAGE expression. The use of RAGE antibody and RNAi to block the AGEs-RAGE pathway resulted in inhibited ERK phosphorylation and cell migration and also reduced expression of MMP2 and MMP9. These findings demonstrate that effects of AGEs on cell migration occur via ERK phosphorylation. Interestingly, a previous clinical study suggested that the increase in RAGE levels in cancer correlated with metastasis [15], [33]. In other cell models, RAGE appeared to regulate cell migration [15], [48], [49]. Our results show that the effects of RAGE RNAi influence cell migration, ERK phosphorylation, and MMP9 expression independently. This finding provides additional support for the contention that RAGE mediates metastasis through the expression of MMP9 via the ERK pathway.

**Figure 6:**
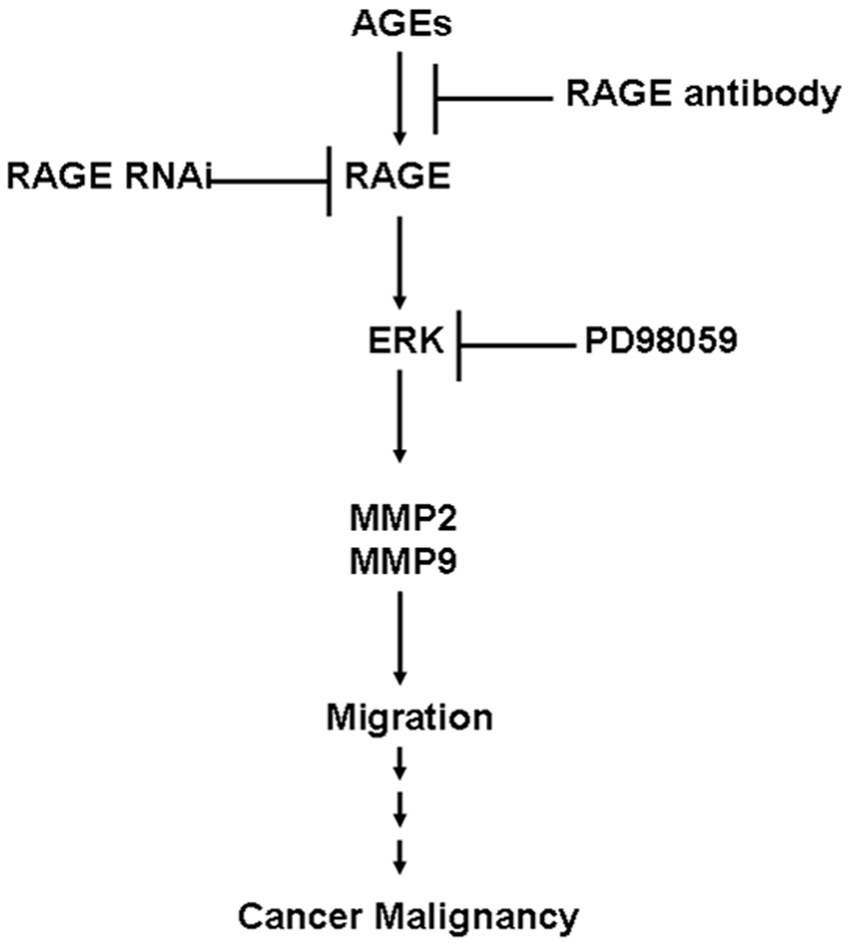
Mechanism by which AGEs-RAGE influences cancer malignancy. AGEs increase RAGE expression and stimulate the downstream pathway of ERK phosphorylation. This has the effect of up-regulating MMP2 and MMP9 expression and enhancing cell migration, which manifests as malignancy.

In this report, AGEs decreased cell viability and enhanced cell migration, while promoting ERK phosphorylation and enhancing the expression of MMP2 and MMP9. PD98059, RAGE antibody, and RNAi were shown to mediate AGE regulation. This is the first study to demonstrate that RAGE regulates the expression of MMP9 via the ERK pathway. More importantly, we demonstrated the role that AGE-RAGE plays in the malignancy of oral cancer through the regulation ERK and downstream pathways, ultimately affecting cell migration in oral cancer. The mechanism by which the AGE-RAGE system functions in our *in vitro* model of oral cancer is presented in Fig. 6. AGEs increase RAGE expression, which stimulates the downstream pathway of ERK phosphorylation and results in the up regulation of MMP2 and MMP9. This in turn enhances cell migration, which manifests in the malignancy of cancer. These findings explain why cases of oral cancer concurrent with DM present increased cancer invasion and reduced survival rates. Results also indicate that the accumulation of AGEs due to aging or DM raises the likelihood of developing malignant cancer.

## Supporting Information

Figure S1 **Closure of cell migration area.** Migration area was measured using ImageJ and quantified by recording changes in the wound area. AGEs significantly decreased the size of wound area, while PD98059, RAGE antibody, and RAGE RNAi suppressed migration at 4 hours. Analysis was conduct by one-way ANOVA, and result was statistically significant (p <0.0001).
(TIF)

Figure S2 **Functionally of MMP 2 and MMP 9.** Following treatment of SAS cells with AGEs (400 µg/ml) for 0.5-4 hours, the expression of ERK phosphorylation was detected. The functionally of MMP 2 and MMP 9 were detected by zymorgraphy following AGEs treatment for 4 or 24 hours. Results show that AGEs increased ERK phosphorylation (A). MMP 2 and MMP 9 were increased by AGEs at 4 hours, but only MMP 2 was increased at 24 hours (B).
(TIF)
