# Supplementary figures and images for: Cell Migration Is Regulated by AGE-RAGE Interaction in Human Oral Cancer Cells In Vitro
Source: PLoS One. 2014 Oct 16;9(10):e110542. doi: 10.1371/journal.pone.0110542 (PMC4199749; doi:10.1371/journal.pone.0110542)

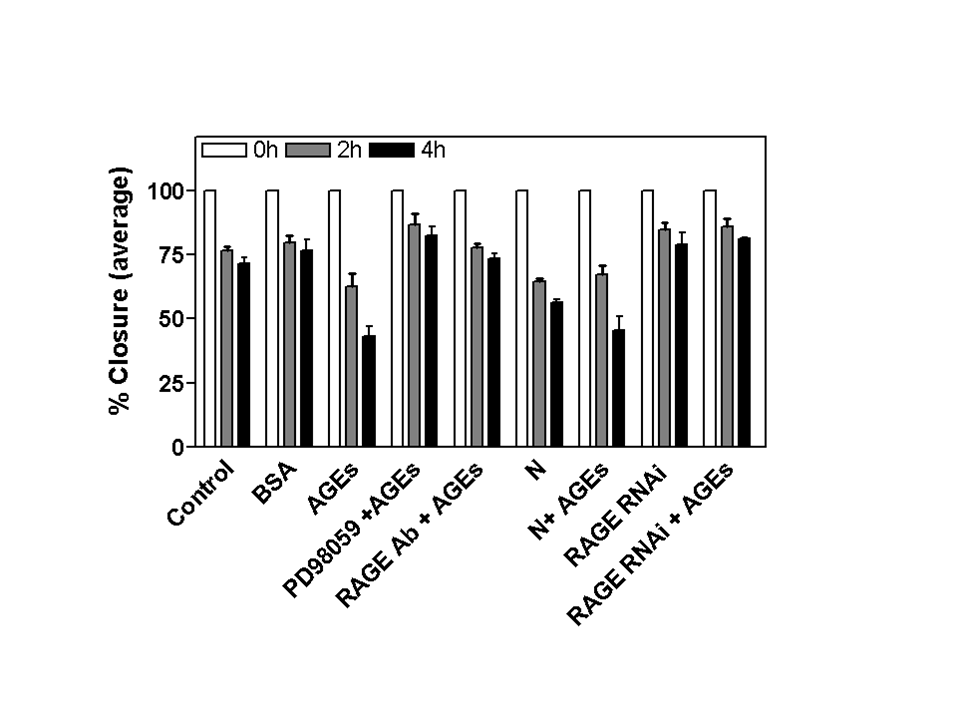

Supplement: Figure S1 — Closure of cell migration area. Migration area was measured using ImageJ and quantified by recording changes in the wound area. AGEs significantly decreased the size of wound area, while PD98059, RAGE antibody, and RAGE RNAi suppressed migration at 4 hours. Analysis was conduct by one-way ANOVA, and result was statistically significant (p <0.0001). (TIF) [file pone.0110542.s001.tif]

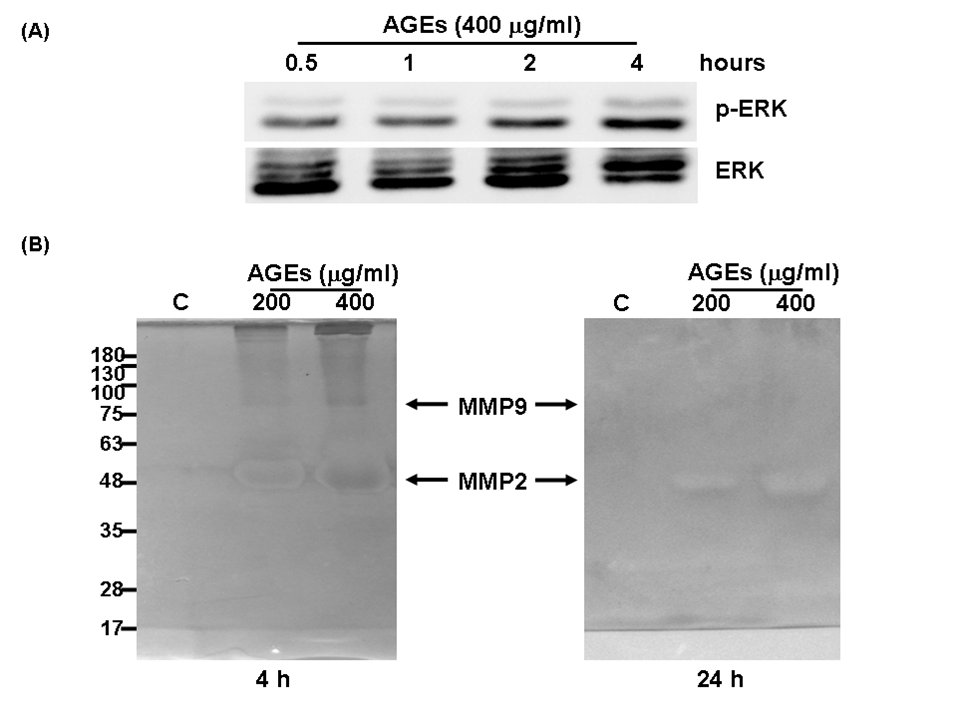

Supplement: Figure S2 — Functionally of MMP 2 and MMP 9. Following treatment of SAS cells with AGEs (400 µg/ml) for 0.5-4 hours, the expression of ERK phosphorylation was detected. The functionally of MMP 2 and MMP 9 were detected by zymorgraphy following AGEs treatment for 4 or 24 hours. Results show that AGEs increased ERK phosphorylation (A). MMP 2 and MMP 9 were increased by AGEs at 4 hours, but only MMP 2 was increased at 24 hours (B). (TIF) [file pone.0110542.s002.tif]
